# Antibiofilm Activity of Acidic Phospholipase Isoform Isolated from *Bothrops erythromelas* Snake Venom

**DOI:** 10.3390/toxins12090606

**Published:** 2020-09-20

**Authors:** Ellynes Nunes, Breno Frihling, Elizângela Barros, Caio de Oliveira, Newton Verbisck, Taylla Flores, Augusto de Freitas Júnior, Octávio Franco, Maria de Macedo, Ludovico Migliolo, Karla Luna

**Affiliations:** 1 Postgraduate Program in Cellular and Molecular Biology—Federal University of Paraíba, João Pessoa, PB 58051-900, Brazil; ellynesnunes@gmail.com (E.N.); taylla.flores@gmail.com (T.F.); gutovfj@dbm.ufpb.br (A.d.F.J.); karlaceatox@yahoo.com.br (K.L.); 2 S-Inova Biotech, Postgraduate Program in Biotechnology—Dom Bosco Catholic University, Campo Grande, MS 79117-010, Brazil; brenoemanuelfarias@gmail.com (B.F.); elihbarros.you@gmail.com (E.B.); ocfranco@gmail.com (O.F.); 3 Protein Purification Laboratory and its Biological Functions, Faculty of Medicine, FAMED, Federal University of Mato Grosso do Sul (UFMS), Campo Grande, MS 79603-011, Brazil; oliveiracfr@gmail.com (C.d.O.); ligiamacedo18@gmail.com (M.d.M.); 4 Embrapa Beef Cattle, Campo Grande, MS 79106-550, Brazil; newton.verbisck@embrapa.br; 5 Center for Proteomic and Biochemical Analysis, Graduate Program in Genomic Sciences and Biotechnology, Catholic University of Brasilia, Brasilia 71966-700, Federal District, Brazil; 6 Center of Biological and Health Sciences, Postgraduate Program in Science and Mathematics Education, Paraíba State University, Campina Grande PB 58429-500, Brazil

**Keywords:** bacterial resistance, animal venom, purification, antibacterial and antibiofilm activity

## Abstract

Introduction: Bacterial resistance is a worldwide public health problem, requiring new therapeutic options. An alternative approach to this problem is the use of animal toxins isolated from snake venom, such as phospholipases A_2_ (PLA_2_), which have important antimicrobial activities. *Bothrops* *erythromelas* is one of the snake species in the northeast of Brazil that attracts great medical-scientific interest. Here, we aimed to purify and characterize a PLA_2_ from *B. erythromelas*, searching for heterologous activities against bacterial biofilms. Methods: Venom extraction and quantification were followed by reverse-phase high-performance liquid chromatography (RP-HPLC) in C18 column, matrix-assisted ionization time-of-flight (MALDI-ToF) mass spectrometry, and sequencing by Edman degradation. All experiments were monitored by specific activity using a 4-nitro-3-(octanoyloxy) benzoic acid (4N_3_OBA) substrate. In addition, hemolytic tests and antibacterial tests including action against *Escherichia* *coli*, *Staphylococcus* *aureus,* and *Acinetobacter* *baumannii* were carried out. Moreover, tests of antibiofilm action against *A. baumannii* were also performed. Results: PLA_2_, after one purification step, presented 31 *N*-terminal amino acid residues and a molecular weight of 13.6564 Da, with enzymatic activity confirmed in 0.06 µM concentration. Antibacterial activity against *S. aureus* (IC_50_ = 30.2 µM) and antibiofilm activity against *A. baumannii* (IC_50_ = 1.1 µM) were observed. Conclusions: This is the first time that PLA_2_ purified from *B. erythromelas* venom has appeared as an alternative candidate in studies of new antibacterial medicines.

## 1. Introduction

With the increase in mortality, morbidity, and the rising demand for spending on diagnostic and therapeutic procedures, public health problems require attention from the scientific community. Among the main pathologies in Brazil and worldwide, nosocomial infections, such as infections caused by bacteria, have become more potent due to the increase in bacterial resistance. These increases are characterized by natural and evolutionary processes observed in microorganisms as responses to environmental stimuli, which are intensified by the incorrect use of antibiotics, leading to bacterial resistance to the usual drugs. In the next 30 years, the number of deaths related to bacterial infections might reach 10 million people worldwide and 392,000 in Latin America alone annually [1,2,3,4].

Bacteria can present two forms of life: the first is planktonic, characterized by independent growth, that is, decoupled from a solid structure or from other organisms such as fungi or even other bacteria, this way of life facilitates their proliferation. The second is as biofilms, which form a community involved in an extracellular matrix composed of several biopolymers, such as extracellular polysaccharides, proteins, DNA, and lipids, in addition to the association of other microorganisms, such as fungi [5,6].

The formation and adhesion of surface bacterial biofilms can be reversible or irreversible, depending on the physical–chemical forces present in the environment. It also depends on the mechanisms of regulation of cell density and collective behavior, called quorum sensing, which allow bacteria to synchronize their gene expression for the formation of the biofilm [7,8,9,10,11,12]. Biofilm formation seems to be related to gene expression and the presence of structures that alter bacterial conformation to the state of biofilm, such as flagella and lashes. The absence of a gene or lack of expression may be directly related to the lack of capacity to form biofilms, even within the same species, depending on different strains [6].

Biofilms provide some benefits to bacteria, including the increased tolerance of such microorganisms to extreme environmental conditions. Furthermore, the exopolysaccharides (EPSs) increase protection against bactericidal agents. This mechanism allows the exchange of genetic material between different species of bacteria and between organisms of the same species, thus facilitating the spread of bacterial resistance, a fact that has aroused interest in the scientific community [12,13,14,15].

On the other hand, the bioprospecting of animal toxin molecules with pharmaceutical application has gained attention, since the variety of these compounds offers alternative candidate sources for the production of new antimicrobial and antitumor drugs for the treatment of viral infections, cancer, and parasitic and bacterial infections [16,17]. Among these sources, snake venoms have a wide variety of components, where about 90% of their dry weight is composed of proteins, among which are phospholipases A_2_ (PLA_2_), enzymatic proteins that generally have low molecular weight. These are responsible for catalyzing the hydrolysis of the 3-sn-phosphoglyceride-dependent calcium 2-acyl ester bond, obtaining lysophospholipids and fatty acid products [18,19,20].

These enzymes play an important role in the metabolism of lipid molecules and are also related to the production and release of arachidonic acid (AA), a precursor of bioactive lipids that participates in cellular activities, due to the release of compounds such as prostaglandins, thromboxane, and leukotrienes, characterizing a perception of pain and inflammation [20,21,22,23,24]. Indeed, bites caused by snakes from the genus *Bothrops* show pharmacological effects characteristic of PLA_2_ action, such as inflammation, local pain, anticoagulant effects, and edema. The viper *Bothrops erythromelas* is of the greatest medical pharmacological interest [25,26].

The A_2_ phospholipases of snake venoms are similar to each other, but they have different toxicological profiles, such as myotoxicity, neurotoxicity, anticoagulant activity, hemolysis, hyperalgesia, inflammation, edema, cytotoxicity, hypotension, and antimicrobial activity [20,27,28,29,30,31]. In this context, the antibacterial activity already observed for phospholipases has drawn attention to the use of these toxins as an alternative for the production of medicines. Therefore, this work aimed to purify A_2_ phospholipases of the venom from *B. erythromelas* and further evaluate their antibacterial and antibiofilm activities.

## 2. Results

### 2.1. Purification and Characterization of PLA_2_ from B. erythromelas

**Figure 1:**
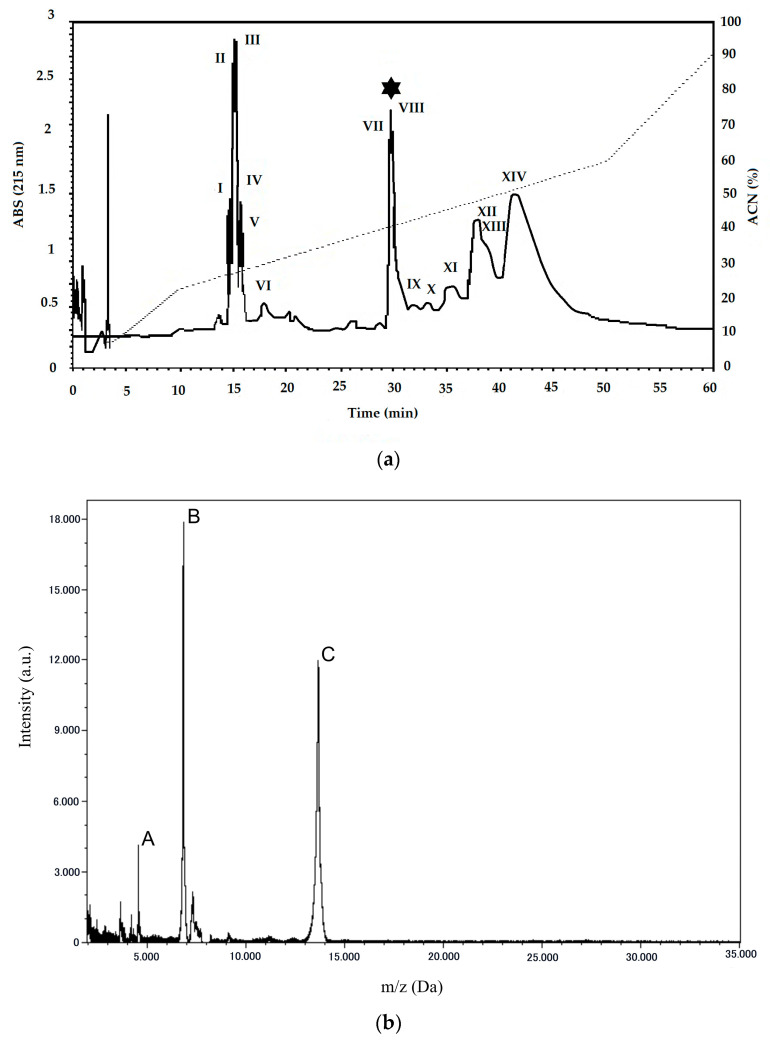
Phospholipase A_2_ (PLA_2_) purification from *Bothrops erythromelas* venom. (**a**) Reverse-phase chromatographic profile, fractions 1 to 14, on a C18 column equilibrated with solvent A (0.1% TFA in water) and eluted with 5–95% solvent B (acetonitrile: solvent A, 9:1, v:v) and a flow rate of 2 mL.min^−1^. (**b**) Fraction 8 (VIII) (*) analyzed by mass spectrometry; ion mass-to-charge ratios are indicated, demonstrating single (C) [M + H]^+^ 13.6564 Da, double (B) [M + 2H]^2+^ 6.8265 Da, and triple (A) [M + 3H]^3+^ 4.5499 Da charge states for the same analyte.

Following venom extraction and lyophilization, the crude venom was applied to a reverse-phase chromatograph (RP-HPLC) using a C18 column. The crude venom exhibited a protein profile of 14 peaks, eluted along the gradient of buffer B (Figure 1a). Peak 8 showed a retention time of 29.4 min and was eluted with ~40% of buffer B, corresponding to the PLA_2_ from *B. erythromelas*.

In order to confirm the purity of the collected PLA_2_, the sample was subjected to analysis in a mass spectrometer (Figure 1b), which generated a spectrum with a mass of 13.6564 Da and the presence of double [M + 2H]^2+^ (6.8265 Da) and triple [M + 3H]^3+^ (4.5499 Da) charge, confirming the purity of the fraction collected from RP-HPLC.

**Figure 2:**
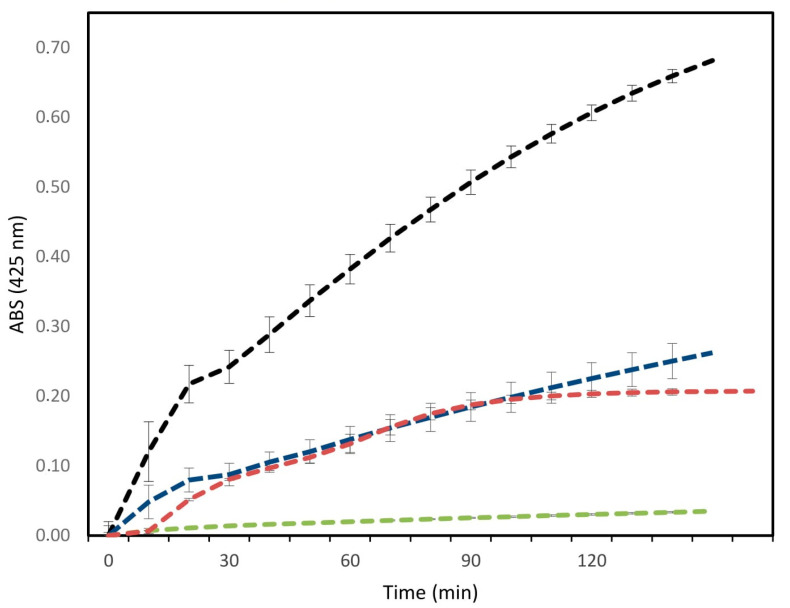
Comparison between activity of the crude venom of *B. erythromelas*, the purified fraction PLA_2_, a commercial phospholipase, and the bovine serum albumin (BSA) consumption of substrate 4N_3_OBA in a concentration of 0.06 µM. Legend colors: black: PLA_2_ isoform; blue: PLA_2_ commercial; red: crude venom; green: BSA.

Figure 2 demonstrates that the PLA_2_ isoform of *B. erythromelas* venom in a concentration of 0.06 µM showed enzymatic activity that was three times more powerful than commercial phospholipase (bovine pancreas phospholipase A_2_—P9913 Sigma) and the crude venom of the snake, compared to the synthetic substrate acid 4-nitro-3-(octanoyloxy)benzoic acid (4N_3_OBA).

Edman’s degradation provided an amino acid sequence with 31 *N*-terminal amino acid residues, with 13 hydrophobic residues and no charge. Subsequently, the sequence was submitted to Basic Local Alignment Search Tool (BLAST), where 96% homology was observed for three acidic PLA_2_: bpPLA_2_-TXI from *Bothrops pauloensis*, sPLA_2_-II from *Bothrops diporus,* and BE-I-PLA_2_ from *B. erythromelas*. To compare the sequences, alignment was performed using ClustalW, where it was possible to observe that only the amino acids Trp^1^ and Asp^25^ in the sequence of our sample are different from the compared sequences, and the PLA_2_ isoform has one hydrophobic residue more than other sequences, configuring a more hydrophobic property for the isoform PLA_2_ (Table 1) [32,33,34].

### 2.2. Hemolytic Activity Assays

Once purified, we investigated the hemolytic activity of PLA_2_ from *B. erythromelas* against murine blood, since the absence of hemolysis is a prerequisite for further biochemical and pharmacological assays. The PLA_2_ from *B. erythromelas* showed no hemolysis when incubated, even at the maximum concentration assayed, from 1.17 to 37.5 µM. This result shows the feasibility for carrying out biological tests with the purified fraction.

### 2.3. Antibacterial and Antibiofilm Activity

The tests showed that the purified PLA_2_ isoform exerts activity in Gram-positive strains. In the first tests with *Staphylococcus aureus* ATCC (American Type Culture Collection) 7133623, there was activity at all concentrations tested, with the best concentration being 37.49 µM, representing 62% ± 17% of activity, whereas for *Escherichia coli* ATCC 25922, low activity was observed at the concentration of 37.49 µM, representing only 12% ± 2% of activity, however, ciprofloxacin showed the best results in the activity observed in PLA_2_, as shown in Table 2 and Figure S1 (Supplementary Materials).

The isolated clinical strain of *Acinetobacter baumannii* 00332126 was then tested. Although it showed greater growth, it also showed better antibacterial activity at concentrations of 37.49 µM, representing 37% ± 10% of activity, showing better activity including compared to antibiotic, which was not able to inhibit bacterial growth of *A. baumannii*. As the *S. aureus* ATCC 7133623 strain did not present biofilm growth, antibiofilm activity was also tested in *A. baumannii* 00332126. The test showed activity at all concentrations through the PLA_2_ isoform. The best concentration was 1.17 µM, with 53% ± 11% of activity, also showing better activity when compared to antibiotic (Table 2 and Figure S1 in Supplementary Materials).

## 3. Discussion

Our results present a PLA_2_ with acidic characteristics that showed a homology of 96% with the only PLA_2_ already described, so far, for *B. erythromelas* venom (BE-I-PLA_2_). The main difference in the purification of our PLA_2_ was in the steps used, because in our work we sought to optimize time by applying only one chromatographic step, i.e., RP-HPLC in a C18 column. In this way, a PLA_2_ was obtained with a molecular mass of 13.6564 Da, whereas for the purification of BE-I-PLA_2_, four steps were applied with different buffers for elution, and column C4 in RP-HPLC, obtaining a PLA_2_ with a molecular mass of 13.64957 Da [34].

The use of several steps during the purification of PLA_2_ from snake venom has been common for a long time. Some studies report the use of at least two stages, such as PLA_2_ isolated from the venom of *Bothrops alternatus*, *Bothrops asper*, and *Bothrops neuwiedi*, using two chromatographic stages, where in the RP-HPLC stages, like us, they used column C18 [35,36,37].

Other reports show the use of up to three stages, such as studies involving the species *Bothrops atrox* and *Bothrops jararaca*, which were subjected to different stages and buffers. The other purifications mentioned were the use of a C18 column in the RP-HPLC stage, differing only from BE-I-PLA_2_ isolated from *B. erythromelas* [34,38,39].

More recent studies point to a reduction in the chromatographic steps during the purification of PLA_2_, as in our work. Using the same methodology applied in the present work, a study involving the species *B. pauloensis* showed the purification of a PLA_2_ (BpPLA_2_-TXI), with 96% homology with our isoform. Confirming that the use of only one step is satisfactory during purification, another study was done with the species *Bothrops cotiara*, which used a methodology similar to ours and managed to purify a basic PLA_2_ with a mass of 13.716 ± 3 Da [32,40].

Therefore, the type of solvent involved in the dilution of the lyophilized sample, as well as the methodology applied, in relation to the linear gradient and the separation column, proved to be important factors for the possible purity of the sample in just one chromatographic step. Thus, it is important to establish the best method of purification, optimizing the time spent on research.

Once purified, we subjected the PLA_2_ from *B. erythromelas* to enzymatic assays. The enzymatic profile observed in Figure 2 demonstrated that our purification process yielded a catalytically active PLA_2_, since a high consumption of the substrate by the purified fraction was observed, indicating a possible Asp^49^ residue, as in the isoform BE-I-PLA_2_ reported earlier [34]. Comparing the enzymatic activity of two PLA_2_ forms isolated from *Bothrops jararacussu* (BthTx-I and BjVIII) with a commercial PLA_2_, and using 4N_3_OBA as substrate, enzymatic activity was observed. This is a basic characteristic of this type of PLA_2_, determining a PLA_2_ Lys^49^. Likewise, the enzymatic activity of a PLA_2_ from *Bothrops neuwiedi urutu*, which contains Lys^49^, was absent when the synthetic substrate 4N_3_OBA was used [37,41]. Similarly, studies using the substrate 4N_3_OBA compared the catalytic activity of a PLA_2_ (Bmaj-9) isolated from the venom of the snake *Bothrops marajoensis* with the crude venom of the same snake. They observed that Bmaj-9 also showed catalytic activity at a concentration of 1.46 µM, higher than that of the snake’s crude venom [42].

The *N*-terminal amino acid sequencing for phospholipase showed two different amino acids when compared with another PLA_2_ (BpPLA_2_-TXI; sPLA_2_-II and BE-I-PLA_2_). However, the similarity among *N*-terminal sequences was maintained at 96%. The presence of a Trp^1^ indicates the greater hydrophobicity of the sample, since this amino acid has aromatic characteristics with a relatively non-polar side chain, which also facilitates the absorption of light. The presence of an Asp^25^ indicates an increase in the acidic characteristic of our sample, since this amino acid is between the two amino acids that have the negatively charged group R at pH 7.0, thus giving it an acidic property. Asp^25^ also justifies the presence of a null charge in the isolated sequence, since the presence of this amino acid increases the positive charges, making them equal to the negative charges, which are consequently annulled [32,33,34,43].

In our study, no hemolytic activity was observed for PLA_2_ from *B. erythromelas*. The lack of hemolytic activity for a PLA_2_ is unusual, but studies speculate that some actions of PLA_2_ are still not well described, based on the absence of toxicity for some prey. Furthermore, the actions may be related to the evolution of this enzyme, which can be present in the venom gland but not develop its expected toxic activity. Studies with an acidic PLA_2_ (BmooPLA_2_) isolated from *Bothrops moojeni* showed a presence of hemolytic activity at 0.07 µM [44].

A further study carried out with an acidic PLA_2_ isolated from *Porthidium nasutum* (PnPLA_2_), displayed hemolytic activity from 0.47 µM [45]. Indirect hemolytic activities in sheep blood were also reported for PLA_2_ from *B. alternatus* at 47.26 µM [35]. However, a study involving the isolation of two PLA_2_ (PLA_2_-12 and PLA_2_-17), from the snake *Micrurus fulvius*, showed that one of the enzymes showed intravascular hemolytic activity in a mice model, namely PLA_2_-17, while PLA_2_-12 did not show intravascular hemolysis in the same model tested [46]. This last result corroborates our findings.

Our findings show that the isoform purified from the *B. erythromelas* venom showed IC_50_ for the Gram-positive strain, whereas for the Gram-negative strains no IC_50_ was reached at the assayed concentration. These data are in accordance with studies where basic the PLA_2_ isolated from *B. marajoensis* venom showed loss of inhibitory activity in all tested strains [47].

The PLA_2_ Lys^49^ from *Lachesis muta* venom, also belonging to the Viperidae family, showed antimicrobial activity against *S. aureus* ATCC 29213 at 0.9 µM. Similarly, studies of a basic PLA_2_ isolated from *Daboia russelii* (Viperidae), showed better antimicrobial effects for Gram-positive bacteria in comparison with that for Gram-negative bacteria [48,49]. These data corroborate our findings.

It is believed that the antimicrobial activity of PLA_2_, especially those with basic properties, is related to disturbances of bacterial membrane integrity [37,50,51]. As Gram-negative bacteria have a cell wall with an outer membrane made up of asymmetric lipids, followed by a layer of peptidoglycans, and an inner membrane made up of phospholipids, it is well established that this conformation makes it difficult for some drugs to enter. This can also be seen in the activity of PLA_2_, since the outer membrane is naturally resistant to the action of PLA_2_ [16].

Otherwise, Gram-positive bacteria have only one layer of peptidoglycans followed by an internal cell membrane, showing that they are more susceptible to the action of a PLA_2_. Thus, the low bactericidal activity of some phospholipases in Gram-negative bacteria compared to the activity observed in Gram-positive bacteria is probably related to the structure of their cell wall, which makes Gram-negative bacteria more resistant to the action of toxic compounds [16,52].

Commercial polypeptide antibiotics, such as bacitracin, act on Gram-positive bacteria, inhibiting the synthesis of the bacterial cell wall, preventing the addition of amino acids and nucleotides to the cell wall. Based on the mechanism of action observed in polypeptide antibiotics, it is believed that such proteins should act similarly to these antibiotics in the tested bacteria [53,54].

Research involving the participation of bioactive molecules from several organisms, such as microalgae, plants, and animals, against biofilms is ongoing. These molecules have several pharmacological and toxicological actions that can be used as an alternative for production of drugs that help in the treatment of infections caused by microorganisms, an emerging problem in the human population, also caused by biofilm formation [55,56].

There are several molecular mechanisms involved in the formation of biofilms between species and between strains of bacteria. It is known that a determining factor for the formation of biofilms is the presence of a disturbance or stress caused by bacteria, as well as the presence of proteins or genes that provide for the formation of these matrices. This is observed in *S. aureus* strains, which have as a determinant for the formation of biofilm the presence of Operon ICA (The intercellular adhesion), or even the formation of a biopolymer essential for the formation of biofilm in this species, such as *N*-acetyl glucosamine [6,57].

In our experiments, however, we observed that the *S. aureus* ATCC 7133623 strain is not capable of forming biofilms. For this is reason, antibiofilm assays were carried out only with *A. baumannii* 00332126.

A study involving the purification of a venom fraction from *Naja ashei*, showed antibiofilm activity against a clinical isolate of *Staphylococcus epidermidis*. In the isolated fraction (F2), the authors report the presence of PLA_2_, in addition to other proteins such as 3FTx and LAAO, they also believe that an antibiofilm activity is performed by PLA_2_, considering that this enzyme is successful for antibacterial activities [58]. This result corroborates with our findings.

Similarly, studies involving an antimicrobial peptide isolated from *Naja atra* (NA-CATH) showed a 50% reduction in the biofilm formation of the bacterium *Burkholderia thailandensis* at a concentration of 0.22 μM, indicating the proven pharmacological potential of snake venoms organic molecules, corroborating the findings, since they have identified a relevant reduction in the biofilm formation of *A. baumannii* 00332126 [59].

The antimicrobial peptide Cath-A, purified from *Bungarus fasciatus* (Elapidae), also reduced *A. baumannii* biofilm formation at ≥2.2 μM. At a higher concentration (≥17.6 µM), Cath-A destroys almost all cells adhering to the biofilm. Further studies with synthetic antimicrobial peptides showed antibiofilm activity against *Pseudomonas aeruginosa* and *A. baumannii* with IC_50_ and IC_90_ 4 and 8 μM, respectively [60,61].

In antibiofilm tests with C-type lectins isolated from *B. jararacussu* venom, an IC_50_ at a concentration of 6.67 µM was observed for *S. aureus* and *S. epidermidis*, but the protein was unable to interfere in bacterial growth [62]. Similarly, a study involving *B. moojeni* isolated molecules showing a reduction in biofilm formation, without influencing bacterial growth [63].

In our studies, growth reduction of the biofilm was obtained from the lowest concentration tested. We observed that the achieved antibiofilm activity was about 20% more concentrated in the enzyme activity of the molecule, indicating a strong interaction between the enzyme and its specific substrate, in view of its low concentration. The reported activity, however, is unusual for acidic PLA_2_, since antibacterial activity is often present in basic PLA_2_, as previously reported. This may explain the activity in biofilm and bacteria at concentrations starting at 20% higher than the enzyme activity of the molecule [47].

This is the first report of an isoform of PLA_2_ that exhibits antibiofilm activity isolated from a snake of the Viperidae family in the literature, demonstrating how molecules from biological sources can contribute to research regarding bacterial infections, acting as an important source of molecules capable of reducing or eradicating biofilms. The PLA_2_ from *B. erythromelas*, purified by our group, is safe for further biological assays, since no hemolytic activity was noticed against murine erythrocytes. These findings emphasize the importance of bioprospection studies with molecules from animal toxins, especially snakes, to control bacterial biofilms, contributing to advances in the control of infections caused by these microorganisms.

## 4. Conclusions

The purification of the PLA_2_ isoform from *B. erythromelas* venom using a single chromatographic step was reported, resulting in protein with 13.6564 Da. The amino-terminal portion of the PLA_2_ isoform showed 96% of identity with another PLA_2_ previously described. Beyond the high enzymatic activity, no hemolytic activity was observed against murine erythrocytes. Notable antibiofilm activity was seen against *A. baumannii* clinical isolates at a low concentration. These findings confirm that purified molecules from snake venoms possess several biological and pharmacological properties. It is therefore necessary to develop basic research around these components, aiming to develop new drugs for the treatment of various diseases that affect human health.

## 5. Methods

### 5.1. B. erythromelas Venom Extraction

*B. erythromelas* venom was collected from 5 adult specimens in captivity at the Zoo for Reptiles of the Caatinga, located in the municipality of Puxinanã, metropolitan region of Campina Grande, state of Paraiba.

After lyophilization, the venom was kept at −20 °C until use. The samples used are registered with the Genetic Heritage Management Council (SisGen) under the register: A883C5B.

### 5.2. Quantification of Venom Proteins

After diluting the lyophilized sample in ultrapure water, the Bradford method (1976), was carried out to quantify the proteins present in the purified fraction. Serial dilutions of the sample were used. As standard for these concentrations, bovine serum albumin (BSA) was used in the same concentration as the purified sample. All samples were tested in triplicate [64].

### 5.3. Purification of Venom Proteins

The crude venom was subjected to high-performance liquid chromatography (Waters and 2695 Separations Module) in a C18 column (Xterra MS 5 µm—4.6 × 250 mm column). The solvent system was composed of 0.1% trifluoroacetic acid (TFA) in H_2_O (Solvent A) and 0.1% TFA in acetonitrile (Solvent B) in a flow of 2 mL.min^−1^ and a linear gradient of 5–95% acetonitrile, for 60 min. Protein peaks were monitored at 216 and 280 nm. The fractions presented in the graphical representation were collected and lyophilized. Subsequently, the fraction with phospholipase activity was selected to be subjected to the mass spectrometer.

### 5.4. Phospholipase Activity

To analyze the phospholipase activity of *B. erythromelas* venom, the methodology described by Holzer and Mackessy (1996) was used, with changes made by Serino-Silva et al. (2014) [65,66].

The substrate for reaction, 4-nitro-3-(octanoyloxy) benzoic acid (4N_3_OBA, Enzy Life Science, Farmingdale, NY, USA) was used. As a positive control, a commercial phospholipase with a concentration of 0.06 µM (1 mg.mL^−1^) (bovine pancreas phospholipase A2—P9913 Sigma) was prepared, and BSA was used as negative protein control, at the same concentration.

### 5.5. Mass Spectrometry

To measure the molecular mass of the selected fraction, a matrix-assisted ionization time-of-flight (MALDI-ToF) mass spectrometer (AutoFlex III) Smartbeam (Bruker Daltonics, Bremen, Germany) controlled by Flex Control 3.0 software was used (Bruker Daltonics, Bremen, Germany). A 0.37 µM sample was solubilized in Ultrapure water, mixed (1:1 v:v) in a saturated solution of sinapinic acid, as matrix, and applied to the target plate (Bruker Daltonics, Bremen, Germany) to dry at room temperature. The compound had its molecular mass obtained in the positive linear mode after external calibration, with Protein Calibration Standard (Bruker Daltonics, Bremen, Germany). The MALDI-ToF spectra were processed with Flex Analysis 3.0 software (Bruker Daltonics, Bremen, Germany).

### 5.6. Amino-Terminal Sequencing of PLA_2_ from B. erythromelas

The amino-terminal sequencing was obtained through Edman’s degradation, using an automatic Shimadzu PPSQ-31B/33B, initially calibrated with the PTH–amino acid mixture standard. A sample of the purified PLA_2_ was resuspended in 37% acetonitrile and applied onto a nitrocellulose membrane (PVDF) and dried under nitrogen flow. According to the manufacturer’s recommendations, phenyl thiohydantoin amino acids were detected after separation on an RP-HPLC C18 column (4.6 × 250 mm). The resulting sequences were applied to the CAST protein BLAST search (BLASTP 2.8.0+) and the significant sequences were aligned using ClustalW 1.2.4.

### 5.7. Hemolysis Test

Erythrocytes of *Mus musculus* were used for the tests, approved by the ethics committee of the Catholic University Dom Bosco (UCDB) under registration no. 014/2018.

The collected blood was stored at 4 °C until use. The cells were washed three times with 50 mM phosphate buffer, pH 7.4. To the erythrocyte suspension was added the fraction of *B. erythromelas* venom referring to phospholipase at a concentration of 0.07 μM, in serial dilution of 1.17 to 37.49 μM in a final volume of 100 mL. The samples were incubated at room temperature for 60 min. After centrifugation at 3000 rpm, hemoglobin release was monitored by reading the absorbance of the supernatant at 425 nm in a SpectraMax microplates readers (Thermo Fisher Scientific Oy, Vantaa, Finland). To control hemolysis, erythrocytes suspended in 5 × 10^4^ µM phosphate buffer, pH 7.4 were used; as a positive control (100% erythrocyte lysis), a 1% (by volume) solution of triton X-100 dissolved in distilled water was used to replace the venom fraction. The tests were performed in triplicate [67].

### 5.8. Antibacterial Activity

Strains of *E. coli* ATCC 25922, *S. aureus* ATCC 7133623, and *A. baumannii* 00332126 (a resistant clinical isolate) were used. For the antibacterial tests, a purified fraction of the venom of the snake *B. erythromelas* with phospholipase activity was used. The tests to observe the antibacterial activity were performed according to the protocol described by CLSI (Clinical and Laboratory Standards Institute), using the 96-well microplate dilution method. Three technical replicates were organized on the microplates at a final bacterial concentration of 2.5 × 10^5^ CFU.mL^−1^ (colony forming unit). The samples were tested in concentrations ranging from 1.17 to 37.49 μM. For positive control, the antibiotic ciprofloxacin was used in the same concentrations as the samples, while the bacterial suspension in MHB (Mueller-Hinton Broth) was used as a negative control [68].

### 5.9. Antibiofilm Activity

Basal Medium 2 (BM2) was used to analyze the biofilm formation. Bacterial cultures of *A. baumannii* 00332126, proven to be clinical isolate resistant, were used. As bacterial suspensions, they were inoculated into 96-well round-bottom plates, including samples from serial dilutions from 1.17 to 37.49 μM. As negative control, only bacteria were used in the BM2 medium, and as a positive control, the antibiotic ciprofloxacin was used in the same concentrations as the sample. To analyze the growth of planktonic cells, an absorbance of 600 nm was used [69,70].

To assess for biofilm formation, performed as described by Naves et al., 2019, the biofilm formation was read at an absorbance of 595 nm. All absorbance readings were performed with the Multiskan GO microplate reader (Thermo Scientific). All tests were performed in triplicate [71].

## Figures and Tables

**Table 1:** Sequence alignment of the phospholipase A_2_ isoform with phospholipase activity with BpPLA_2_-TXI, sPLA_2_-II, and BE-I-PLA_2_, using the ClustalW tool. Legend: asterisk = identity.

Species	Access Number	PLA_2_	Alignment	Homology (%)	Charge
*B. erythromelas*	-	PLA_2_ Isoform	WLVQFETLIMKIAGRSGVWYYGSYDCYCGSG	-	0
*B. pauloensis*	D0UGJ0.1	BpPLA_2_-TXI	NLVQFETLIMKIAGRSGVWYYGSYGCYCGSG	96	+1
*B. diporus*	AFJ79208.1	sPLA_2_-II	NLVQFETLIMKIAGRSGVWYYGSYGCYCGSG	96	+1
*B. erythromelas*	Q2HZ28.1	BE-I-PLA_2_	SLVQFETLIMKIAGRSGVWYYGSYGCYCGSG	96	+1
			***********************.******		

The similarity observed in the purified fraction with the phospholipases BpPLA_2_-TXI, sPLA_2_-II, and BE-I-PLA_2_ offers reliable indications of an acidic characteristic in our sample.

**Table 2:** Antibacterial and antibiofilm activity and IC_50_ in vitro evaluation for PLA_2_ against *Escherichia coli* ATCC 25922, *Acinetobacter baumannii* 00332126, and *Staphylococcus aureus* ATCC 7133623 compared to ciprofloxacin antibiotic activity.

Bacteria	Concentration (µM)	Ciprofloxacin (%)	Activity PLA_2_ (%)	IC_50_ PLA_2_ (µM)
*E. coli* ATCC 25922	37.4	97 ± 16	12 ± 20	-
*S. aureus* ATCC 7133623	37.4	90 ± 18	62 ± 17	30.2
*A. baumannii* 00332126	37.4	0	37 ± 10	-
**Biofilm**				
*A. baumannii* 00332126	1.17	7 ± 6	53 ± 11	1.1

## Supplementary Materials

The following are available online at https://www.mdpi.com/2072-6651/12/9/606/s1, Figure S1: Antibacterial and antibiofilm activity of the PLA2 isoform.
