# Supplementary material for: Antibiofilm Activity of Acidic Phospholipase Isoform Isolated from Bothrops erythromelas Snake Venom
Source: Toxins (Basel). 2020 Sep 20;12(9):606. doi: 10.3390/toxins12090606 (PMC7551604; doi:10.3390/toxins12090606)
Supplement: Supplementary file 1 [file toxins-12-00606-s001.pdf]

# Supplementary Materials: Antibiofilm Activity of Acidic Phospholipase Isoform Isolated from *Bothrops erythromelas* Snake Venom

**Ellynes Nunes, Breno Frihling, Elizângela Barros, Caio de Oliveira, Newton Verbisck, Taylla Flores, Augusto de Freitas Júnior, Octávio Franco, Maria de Macedo, Ludovico Migliolo and Karla Luna**

(a)

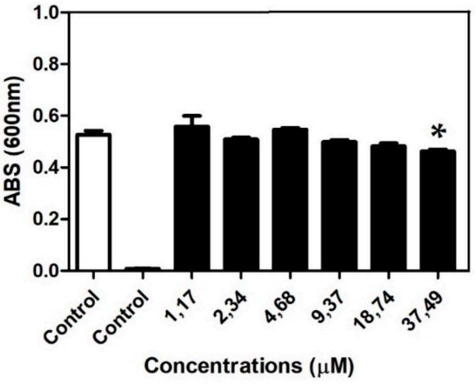


(b)

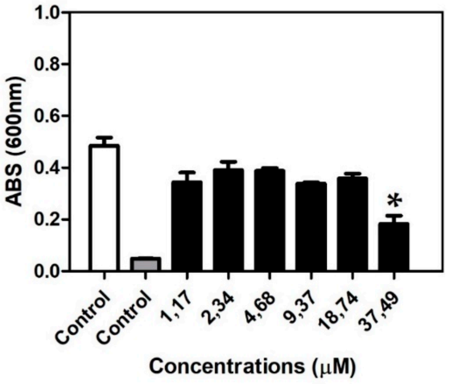


(c)

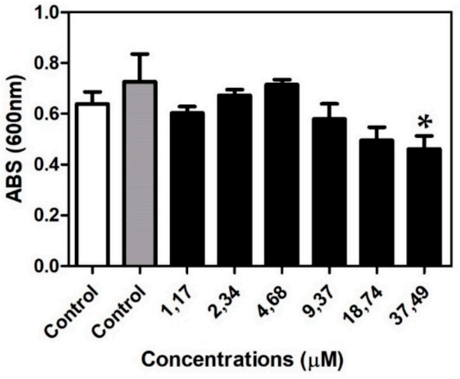


(d)

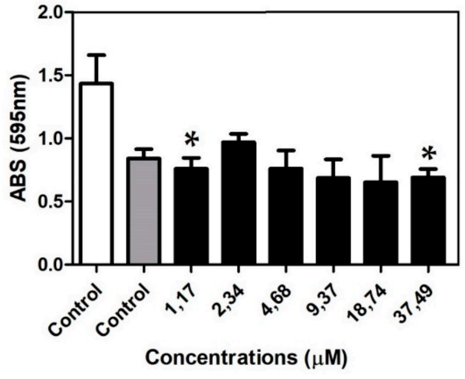


**Figure S1**: Antibacterial and antibiofilm activity of the $PLA_2$ isoform. (**a**) Antibacterial activity against the *E. coli* strain. (**b**) Antibacterial activity against the *S. aureus.* (**c**) Antibacterial activity against the A. *baumannii.* (**d**) Antibiofilm activity against *A. baumannii*. White bars correspond to negative control; gray bars correspond positive control and black bars to $PLA_2$ isoform
